# Developmental origins of variability in pelvic dimensions: Evidence from nulliparous South Asian women in the United Kingdom

**DOI:** 10.1002/ajhb.23340

**Published:** 2019-11-22

**Authors:** Meghan K. Shirley, Tim J. Cole, Owen J. Arthurs, Chris A. Clark, Jonathan C.K. Wells

**Affiliations:** ^1^ UCL Great Ormond Street Institute of Child Health London UK; ^2^ School of Public Health University of São Paulo São Paulo Brazil; ^3^ Department of Radiology Great Ormond Street Hospital London UK

## Abstract

**Objectives:**

Pelvic growth may be sensitive to early‐life nutrition, with implications for maternal risk of obstructed labor. However, the “developmental origins” of adult pelvic variability require further investigation. We tested whether adult pelvic dimensions are associated with two components of height, indexing different periods of linear growth: tibia length, a proxy for early postnatal growth, and height‐residual (height regressed on tibia length), a proxy for later growth. We also tested whether adult pelvic dimensions are associated with birth weight, a marker of nutritional investment in utero.

**Methods:**

In this cross‐sectional study, data were obtained on 68 nulliparous young women of South Asian ancestry. Pelvic dimensions (bi‐iliac and bi‐acetabular breadth, anteroposterior pelvic inlet and outlet, interspinous and intertuberous diameter) were measured using magnetic resonance imaging. Height and tibia length were measured manually. Birth weight and gestational age were obtained by recall. Multivariable regression models were fitted with a given pelvic dimension regressed on height‐residual, tibia, and birth weight, with the latter adjusted for gestational age.

**Results:**

Controlling for birth weight, height‐residual was predictive of bi‐acetabular breadth, bi‐iliac breadth, and the pelvic inlet, while tibia length significantly predicted all dimensions except interspinous diameter. Controlling for the linear growth variables, birth weight was predictive of bi‐iliac breadth only.

**Conclusions:**

Markers of linear growth during both early and later development were associated with adult pelvic dimensions, whereas size at birth was poorly predictive. Efforts to reduce stunting in early life may facilitate the attainment of maximum potential growth for both height and the pelvis.

## 1 INTRODUCTION

Obstructed labor and its associated conditions represent a significant source of maternal and infant morbidity and death, particularly in low‐ and middle‐income countries (Konje & Ladipo, 2000; Mselle, Moland, Evjen‐Olsen, Mvungi, & Kohi, 2011; Ronsmans et al., 2006). In some populations, fatal complications including hemorrhage, infection, and asphyxia, contribute to mortality rates similar to those in high‐income countries in the early 20th century (Goldenberg & McClure, 2015). Global disparities in morbidity/mortality associated with obstructed labor may reflect both geographical genetic variation (Betti & Manica, 2018; Camilleri, 1981) and unequal access to health care (Ronsmans et al., 2006) but maternal phenotypic variability related to environmental variability also merits attention.

Risk for operative delivery (eg, cesarean section) due to obstructed labor has long been associated with variation in maternal height (Baird, 1949, 1965; Konje & Ladipo, 2000; Merchant, Villar, & Kestler, 2001; Mogren et al., 2018; Stulp, Verhulst, Pollet, Nettle, & Buunk, 2011; Van Roosmalen & Brand, 1992). Shorter women are more likely to experience disproportion between dimensions of the pelvic canal and fetal body (Aitken & Walls, 1986; Toh‐adam, Srisupundit, & Tongsong, 2012; Tsu, 1992), despite some evidence for a protective effect of maternal height‐pelvic shape covariance (Fischer & Mitteroecker, 2015; Kurki, 2007). Adult height of women has been linked to their pelvis size in several studies (Bernard, 1952; Cox, 1963; Holland, Cran, Elwood, Pinkerton, & Thompson, 1982; Kakoma, 2016; Sharma, Gupta, & Shandilya, 2016), although others have reported more complex associations (Tague, 2000), or no association (Pan, 1929). With respect to fetal size, most attention has been directed to the head; however, components of birth size such as weight and shoulder width are also relevant (Merchant et al., 2001; Stulp et al., 2011; Trevathan & Rosenberg, 2000).

Global variation in the incidence of feto‐maternal disproportion is likely influenced by maternal and fetal nutrition and growth, both within and across generations (Konje & Ladipo, 2000; Wells, 2017). Adult height is the outcome of growth in preadult life, which is both shaped by genetics and responsive to environmental stimuli through plasticity (ie, nongenetic phenotypic adaptation within the life course; Tanner, 1992; Bailey et al., 2007; Victora et al., 2008; Cameron, 2012). Fetal life and infancy are primary periods of human growth plasticity (Lejarraga, 2012). Short adult stature may reflect poor nutritional experience during these periods, influenced by a range of environmental factors including dietary intake, stress, food quality, and infectious disease burden (Martorell, 1989; NCD‐RisC, 2016; Steckel, 1995; Stinson, 2012). Prior work has suggested the pelvis may be similarly affected by such environmental exposures, in addition to genetic factors (Abitbol, 1996; Angel, Kelley, Parrington, & Pinter, 1987; Fischer & Mitteroecker, 2015; Kelley & Angel, 1987; Nicholson, 1945; Sharma, 2002; Thoms, 1936, 1947). Building on this literature, we examine the pelvis in the context of the “developmental origins of health and disease” (DOHaD), a life‐course approach, which sheds light on associations of fetal and postnatal development during infancy and early childhood with later ill‐health (Agarwal et al., 2018; Barker, 1990; Emanuel, 1986; Hales & Barker, 1992; Kermack, McKendrick, & McKinlay, 1934; McMillen & Robinson, 2005; Wadsworth, 1997).

Recruiting a cohort of young, nulliparous women and using a cross‐sectional study design, we tested whether adult pelvic dimensions measured by magnetic resonance imaging (MRI) are associated with adult height, and with markers of three specific growth periods. Birth weight served to index nutritional investment in fetal life. Tibia length and height‐residual (height minus tibia length) were used to index linear growth in the first 2 to 3 years of life, and linear growth from ~3 years to its cessation, respectively. We used tibia length following previous authors (Bailey et al., 2007; Pomeroy et al., 2012), as leg length has been shown to be more sensitive than trunk length to early‐life ecological circumstances (Bogin & Baker, 2012; Sanna & Soro, 2000), and the length of the tibia may be particularly sensitive (Jantz & Jantz, 1999; Holliday & Ruff, 2001). Although necessarily an approximation, we assigned tibia length to index linear growth from birth to ~2 to 3 years because in this period growth is relatively rapid, highly sensitive to the environment, and adverse experience may confer lasting effects on tibial phenotype (Cameron, 2012; Cole, 2000a; Lejarraga, 2012; Martorell, 1989). Linear growth is relatively canalized after this period (ie, less subject to environmental perturbations), with greater influence exerted by genes regulating adult size (Cameron, 2012; Lejarraga, 2012; Martorell, 1989). We can then consider height‐residual, which is statistically independent of tibia length, to be an index of this less environmentally sensitive growth period.

We recruited South Asian women living in London, UK, for a larger study of variation in body composition, including the pelvis, and resting energy expenditure (Shirley et al., 2018). South Asian populations are among the shortest worldwide (NCD‐RisC, 2016), and despite improvements in maternal and child health, continue to experience high rates of maternal and newborn death from obstructed labor and intrapartum‐related events, among other causes (Akseer et al., 2017).

## 2 MATERIALS AND METHODS

Participants were included based on the following criteria: they were healthy; female; nulliparous; of South Asian ancestry; aged between 20 and 28 years; and had a body mass index (BMI) in the range 17 to 28 kg/m^2^. Ancestry was ascertained by subject self‐identification, and participants confirmed that their maternal and paternal grandparents were South Asian. Subjects traced ancestry to, but were not necessarily born in, four target countries: India, Pakistan, Bangladesh, and Sri Lanka. The age range was chosen to avoid phenotypic variability associated with pubertal growth and aging, while nulliparous condition avoided confounding by differential parity. The BMI criterion sought to exclude very underweight, very overweight, or obese women, so as to focus on those of normal body weight. We shifted our BMI range to the left of the WHO's typical classification because, in general, Asian populations demonstrate lower mean or median BMI compared to non‐Asian populations (WHO Expert Consultation, 2004). Exclusion criteria were health conditions potentially impacting growth, skeletal deformity of the tibia or pelvis, smoking, and contraindications for MRI.

Methods of recruitment included posters, online advertisements, and word‐of‐mouth. Data collection took place from March 2015 to May 2016 at the UCL Great Ormond Street Institute of Child Health and Great Ormond Street Hospital for Children NHS Trust. All participants attended 1 to 2 appointments to complete measurements. The larger study of body composition and metabolic variability from which the current data were derived received ethical approval from a Research Ethics Committee of the NHS Health Research Authority. All participants gave written, informed consent.

Height was measured in duplicate to the nearest 0.1 cm using a wall‐mounted stadiometer (Holtain). Weight was measured in duplicate to the nearest 0.01 kg using a scale integral to the BodPod air‐displacement plethysmography system (Cosmed, Rome, Italy). Large sliding calipers (Harpenden) were used to measure in duplicate, to the nearest millimeter, tibia length as the distance from the medial tibial plateau to the inferior edge of the medial malleolus on the left leg only, following standard protocols (Pomeroy et al., 2012). We obtained information on birth weight and gestational age by subject recall (more specifically, based on subjects' parents' recall, or birth records held by the family).

High‐resolution 3D body imaging was undertaken using a 3T Siemens Magnetom Prisma scanner. We performed a volumetric 3D T_2_‐weighted acquisition of the pelvis using 144 contiguous coronal slices (TR 15.5 ms, TE 5.1 ms, flip angle 25°, voxel size 1.2 × 1.2 × 1.2 mm, 1 average; scan duration ~5 minutes).

**Figure 1:**
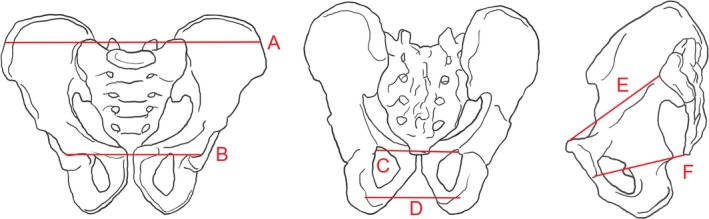
Locations of pelvic measurements, as measured on MR images. Pelvic non‐canal (A, bi‐iliac breadth; B, bi‐acetabular breadth) and canal (C, interspinous diameter; D, intertuberous diameter; E, inlet anteroposterior; F, outlet anteroposterior) measurements. Images adapted from Kurki and Decrausaz (2016)

Six pelvic dimensions (bi‐iliac breadth, bi‐acetabular breadth, anteroposterior dimensions of the inlet and outlet, interspinous diameter, and intertuberous diameter) were measured from raw DICOM images using OsiriX open‐source software (v8.5; Rosset, Spadola, & Ratib, 2004). Straight lines were drawn between anatomical landmarks for each of the six pelvic outcomes as described in Table 1 and as shown in Figure 1.

**Table 1:** Descriptions of MRI pelvic measurements

Measurement	Description
Bi‐acetabular breadth^a^	Distance between the acetabula
Bi‐iliac breadth^a^	Maximum breadth across the iliac blades
Pelvic inlet anteroposterior	Anteroposterior distance from the most superior aspect of the pubic symphysis to the sacral promontory
Pelvic outlet anteroposterior	Anteroposterior distance from the most inferior‐medial aspect of the pubic symphysis to the tip of the coccyx
Interspinous diameter	Distance between the tips of the ischial spines, measured on axially‐oriented MR image
Intertuberous diameter	Distance between ischial tuberosities at their most posterior aspect, measured on axially‐oriented MR image

Abbreviation: MRI, magnetic resonance imaging.

a Definitions from Kurki (2007).

All other definitions from Salerno, Daniels, Brown, Heald, and Moran (2006) and Hong et al. (2009).

Duplicate measurements of each pelvic dimension were taken by the first author (MKS) on different days and averaged for analysis. A small number of measurements were considered unreliable due to reduced image quality and were thus excluded. The anteroposterior pelvic outlet measure was particularly affected. This is reflected in the table containing descriptive statistics and sample size for each measure (see below).

## 3 STATISTICAL ANALYSIS

Birth weight SD scores (SDS) were derived from birth weight and gestational age using UK‐WHO reference birth centiles (Cole, Williams, & Wright, 2011). Height, tibia, and pelvis dimensions scale allometrically with one another (Fischer & Mitteroecker, 2017; Meadows & Jantz, 1995), thus these variables were natural log‐transformed for analysis. Residuals generated by the regression of log height on log tibia were used as a “height‐residual” variable, an index of growth variability independent of tibia length. Pearson correlations were calculated for pelvic dimensions with height, height‐residual, tibia length, and birth weight SDS. Data were plotted to visualize associations of pelvic dimensions with growth predictors.

A set of multivariable regression models was fitted with a given pelvic measure as the dependent variable, and height‐residual, tibia, and birth weight SDS included as independent predictors. Beta coefficients indicated the predicted percentage increase in a given pelvic dimension associated with a 1% increase in a given growth predictor, controlling for the others (Cole, 2000b). An indicator variable flagging those born outside India, Pakistan, Bangladesh or Sri Lanka was added to the models as a potential confounder. All statistical analyses were carried out using the R language for statistical computing (v3.5.2; R Core Team, 2018) in RStudio (v1.1.463) with two‐tailed significance tests at α = 0.05.

## 4 RESULTS

Seventy female participants were recruited. Most were students attending university in or near London, UK. Fifty‐one percent of them reported Indian ethnicity, 11% Pakistani, 11% Bangladeshi, and 11% Sri Lankan, while 13% reported mixed ancestry among the four represented countries. One participant's ancestors had emigrated to Mauritius from India. Forty‐seven percent of the sample was born in South Asia, while the majority of subjects born elsewhere were born in the United Kingdom. The measured BMI range was 17 to 30 kg/m^2^, with two subjects misreporting weight and height at recruitment. One reported gestational age was 34 weeks while the others ranged from 37 to 42 weeks. Two subjects missed the MRI pelvis measurement, resulting in a final sample size of 68.

Mean absolute differences between repeated pelvic measurements were all <1.5% except pelvic outlet (3.2%) and interspinous diameter (1.6%). Table 2 provides descriptive statistics for the sample, along with sample size and coefficients of variation (CV) for pelvic dimensions. CV was highest for pelvic outlet and lowest for bi‐acetabular breadth; CVs for the other four dimensions were very similar.

**Table 2:** Descriptive statistics and coefficients of variation for pelvic dimensions

Subject characteristic	n	Mean ± SD	Range	CV%
Age (y)	70	24 ± 2.4	20 to 28	
Birth weight (kg)	69	3.15 ± 0.5	2.0 to 4.5	
Gestational age (wk)	67	39.3 ± 1.5	34 to 42	
Birth weight SDS	67	−0.43 ± 1.1	−2.9 to 2.4	
Height (cm)	70	161 ± 6.6	148 to 177	
Weight (kg)	70	57.8 ± 9.2	40.7 to 81.1	
BMI (kg/m^2^)	70	22.2 ± 3.5	17.2 to 30.3	
Tibia length (cm)	70	36.8 ± 2.5	30.0 to 42.9	
Pelvic measurements (cm)				
Bi‐acetabular breadth	68	13.3 ± 0.7	11.9 to 14.7	5.3
Bi‐iliac breadth	68	25.1 ± 1.7	20.5 to 28.3	6.8
Pelvic inlet anteroposterior	67	12.9 ± 0.9	10.6 to 15.2	7.0
Pelvic outlet anteroposterior	58	8.9 ± 0.9	7.1 to 10.8	10.1
Interspinous diameter	66	10.9 ± 0.7	9.6 to 12.6	6.4
Intertuberous diameter	67	13.3 ± 0.9	11.1 to 15.0	6.8

Abbreviations: CV, coefficient of variation; MRI, magnetic resonance imaging; SDS, standard deviation score.

Correlations among pelvic measurements, total height, and growth‐period proxy variables (height‐residual, tibia length, and birth weight SDS) are shown in Table 3. All pelvis dimensions were significantly correlated with height, where pelvic inlet and bi‐iliac breadth demonstrated the largest coefficients, and interspinous diameter the smallest. Height‐residual correlated with bi‐acetabular breadth, bi‐iliac breadth and pelvic inlet, but not with pelvic outlet or interspinous diameter; its correlation with intertuberous diameter was borderline significant. Tibia length correlated significantly with all pelvic dimensions except interspinous diameter. Birth weight SDS was associated with bi‐acetabular and bi‐iliac breadths; it was not associated with height.

**Table 3:** Pearson correlation coefficients of MRI pelvic measurements with height, height‐residual, tibia length, and birth weight SDS^a^

Pelvic dimensions (cm)	Height (cm)	Height‐residual (cm)	Tibia (cm)	BW SDS
Bi‐acetabular breadth	0.44 (0.23, 0.61)	0.42 (0.20, 0.60)	0.28 (0.05, 0.49)	0.27 (0.03, 0.48)
Bi‐iliac breadth	0.52 (0.33, 0.68)	0.45 (0.24, 0.62)	0.36 (0.13, 0.55)	0.40 (0.17, 0.59)
Pelvic inlet AP	0.59 (0.40, 0.72)	0.45 (0.24, 0.63)	0.43 (0.21, 0.61)	0.08 (−0.17, 0.32)
Pelvic outlet AP	0.35 (0.10, 0.56)	0.21 (−0.05, 0.45)	0.28 (0.03, 0.50)	0.06 (−0.20, 0.32)
Interspinous diameter	0.27 (0.03, 0.48)	0.20 (−0.04, 0.42)	0.21 (−0.04, 0.43)	0.13 (−0.13, 0.36)
Intertuberous diameter	0.41 (0.18, 0.59)	0.24 (−0.00, 0.45)	0.34 (0.11, 0.53)	0.18 (−0.07, 0.41)

Abbreviations: AP, anteroposterior; BW SDS, birth weight SD score; MRI, magnetic resonance imaging.

a Values are *r*, 95% CI; height‐residual is height adjusted for tibia length; pelvic dimensions, height, and tibia log transformed.

The ability of birth weight, tibia, and height‐residual to act as markers of distinct growth periods depended on their mutual independence. Height‐residual was by definition unrelated to tibia, while birth weight SDS was unrelated to both height‐residual (*r* = 0.20, *P* = .1) and tibia (*r* = 0.003, *P* = .9).

**Figure 2:**
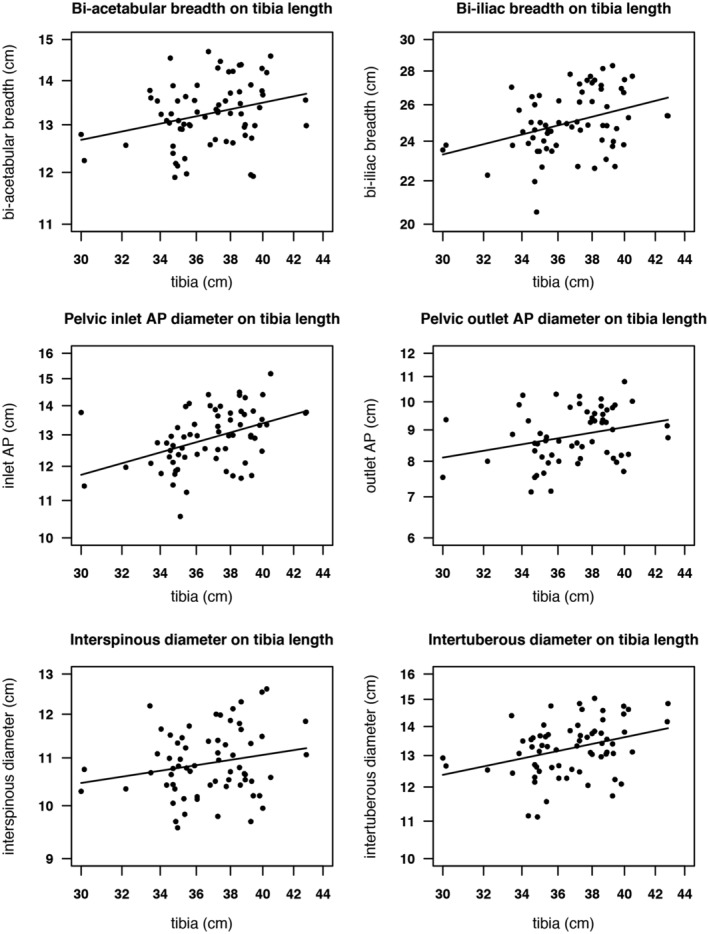
Plots of pelvic dimensions against tibia length. All variables are log transformed; lines are fitted least‐squares regressions. AP, anteroposterior

**Figure 3:**
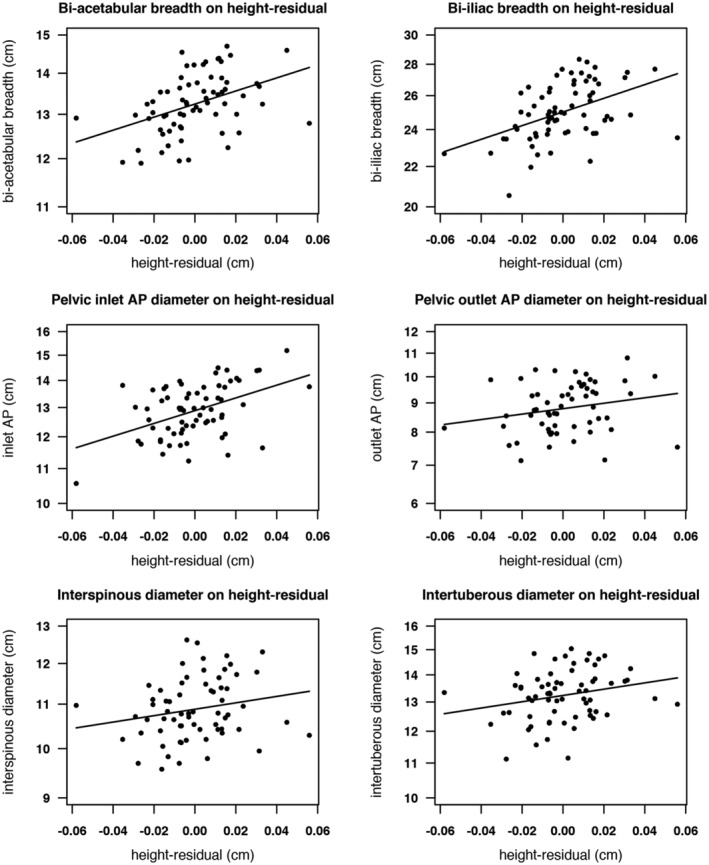
Plots of pelvic dimensions against height‐residual. Pelvic dimensions are log transformed; lines are fitted least‐squares regressions. AP, anteroposterior

Plots of pelvis dimensions against tibia length are shown in Figure 2, while pelvis dimensions against height‐residual are shown in Figure 3. Scaling each dimension to fit the *y*‐axis means that the slopes of the regression lines reflect the relative correlations in Table 3.

Multivariable regression models of pelvic dimensions on height‐residual, tibia length, and birth weight SDS are given in Table 4. Controlling for the other variables, height‐residual was a significant predictor of bi‐acetabular breadth, bi‐iliac breadth, and pelvic inlet, while tibia length significantly predicted all pelvic dimensions except interspinous diameter. Controlling for the two linear growth variables, birth weight SDS significantly predicted bi‐iliac breadth only. In the models where bi‐acetabular breadth, bi‐iliac breadth, or pelvic inlet were entered as dependent variables, the three independent variables explained 26%, 40%, and 36% of the variance, respectively.

**Table 4:** Multivariable regressions of pelvic measures on height‐residual, tibia length, and birth weight SDS^a^

Pelvic dimensions (cm)	Growth predictors						Adj. *R* ^2^
	Height‐residual (cm)		Tibia (cm)		BW SDS		
	b	95% CI	b	95% CI	b	95% CI	
Bi‐acetabular breadth	1.02	0.40, 1.63	0.23	0.06, 0.40	0.01	−0.00, 0.02	0.26
Bi‐iliac breadth	1.37	0.66, 2.08	0.36	0.16, 0.55	0.02	0.01, 0.03	0.40
Pelvic inlet AP	1.79	0.99, 2.58	0.45	0.23, 0.67	−0.00	−0.02, 0.01	0.36
Pelvic outlet AP	1.07	−0.30, 2.45	0.43	0.05, 0.80	−0.00	−0.03, 0.03	0.08
Interspinous diameter	0.51	−0.35, 1.37	0.17	−0.06, 0.41	0.01	−0.01, 0.02	0.03
Intertuberous diameter	0.76	−0.09, 1.61	0.28	0.05, 0.52	0.01	−0.01, 0.02	0.12

Abbreviations: AP, anteroposterior; BW SDS, birth weight SD score; CI, confidence interval.

a Each row is a single regression model with three predictors (height‐residual, BW SDS, and tibia); height‐residual is height adjusted for tibia length; pelvic dimensions and tibia log transformed.

## 5 DISCUSSION

An early study by Pan (1929) found no association between height and pelvic dimensions in Indian women. Our results, however, are in line with a number of subsequent studies, which reported positive associations between adult height and the pelvis (Bernard, 1952; Cox, 1963; Holland et al., 1982; Kakoma, 2016; Sharma et al., 2016). To investigate the impact of earlier vs later linear growth on pelvis size, we partitioned height into tibia length, which is suggested to be a sensitive marker of early‐life environmental conditions and thus may act to index growth in this period (Bailey et al., 2007; Pomeroy et al., 2012), and height‐residual. The latter, a component of height variability independent of tibia length and including the thigh (femur) and trunk, served to index growth after the first 2 to 3 years of life. We found that both components of height significantly predicted several pelvic dimensions, and the correlations of tibia or height‐residual with pelvic outcomes were similar (see Figures 2 and 3). This suggests that linear growth in both earlier and later periods is important for adult pelvis size. In contrast, birth weight, our index of nutritional investment in fetal life, was a significant predictor of only one pelvic dimension, bi‐iliac breadth.

With respect to the relative variability of the pelvic dimensions investigated, the anteroposterior outlet had the highest CV. For example, its value of 10.1% contrasted with that of the intertuberous diameter (6.8%), which is a transverse measure of the outlet. It is possible that the anteroposterior outlet's relatively high variability in our sample is related to variation in sacral/coccygeal length (Tague, 2017), however, greater measurement error for this dimension due to poor image quality around the coccyx is also plausible.

Several variables could have served as linear growth proxies. Because tibia and femur lengths are likely highly correlated, height‐residual may include some tibia information via the femur. If we used leg length (tibia + femur) as our proxy of early linear growth, however, height‐residual would represent sitting height, and be far less predictive without the femur component. Authors have often used relative leg length (leg length/height) to index early childhood environmental experience (eg, Bogin, Smith, Orden, Varela‐Silva, & Loucky, 2002; Lawlor, Taylor, Davey Smith, Gunnell, & Ebrahim, 2004), however, the use of tibia or lower leg length for this purpose is similarly well supported (but see Kinra, Rameshwar Sarma, Hards, Davey Smith, & Ben‐Shlomo, 2011). In a study from Peru, for example, tibia length showed greater differences than head‐trunk height or foot length between differentially stressed highland and lowland children (Pomeroy et al., 2012). Similarly, Bailey et al. (2007) used differential growth of the tibia to assess the effects of variation in nutrition and susceptibility to hypoxic stress in Han and Tibetan children at high altitude. In Mexican Maya children, knee height as a measure of distal leg length (indexing the tibia) associated more strongly with environmental variables than with those reflecting ancestry (Vázquez‐Vázquez et al., 2013). Bogin and Varela‐Silva (2008) suggested that “measuring lower leg length is especially important as the tibia is more sensitive to changes in the health environment than is the femur or the long bones of the arm” (p. 207).

At the same time, tibia growth in absolute terms also reflects fetal experience, demonstrated by the detrimental impacts of maternal smoking (Lindsay, Thomas, & Catalano, 1997), diabetes (Lampl & Jeanty, 2004), and intrauterine growth restriction (Goetzinger, Cahill, Macones, & Odibo, 2012) on leg length. It is therefore more appropriate to consider birth weight a marker of in utero nutritional supply, rather than growth per se, whereas tibia length and height‐residual are more direct growth measures. Importantly, though, we have shown that birth weight SDS is not associated with tibia length in our sample, allowing these variables to function as independent markers of fetal and postnatal life in the present analysis.

The lack of association between birth weight and most pelvic dimensions was unsurprising, considering the lack of association between birth weight and height, while height does correlate with the pelvis. However, the fact that birth weight did not correlate with height is itself surprising in the light of previous findings (Adair, 2007; Haeffner, Barbieri, Rona, Bettiol, & Silva, 2002; Sachdev et al., 2005; Victora et al., 2008). In Delhi, India, for example, increased birth weight and birth length were associated with increased adult height (Sachdev et al., 2005), and a positive birth weight‐adult height relationship was observed in pooled results from study cohorts in Brazil, Guatemala, India, South Africa, and the Philippines (Victora et al., 2008). Our findings may be explained by poor sensitivity of birth weight as a fetal growth marker, for example, resulting from noise in the data due to variability in fat mass. Fat mass as a component of birth weight is not only highly variable, but also apparently increased relative to lean mass in South Asians at birth (Modi et al., 2009; Yajnik et al., 2003).

Beyond this, birth weight and gestational age may have been noisy due to poor recall. In order to explore the level of precision in birth weights required for our findings to stand, we conducted a sensitivity analysis based on the UK study of O'Sullivan and colleagues. The authors found in a large sample that 97% of parents recalled their child's birth weight within 500 g of hospital records, up to 16 years after delivery (O'Sullivan, Pearce, & Parker, 2000). In a single analysis, we substituted our collected birth weight values with values randomly increased or decreased by 500 g. Using the adjusted values, correlations of birth weight SDS with bi‐iliac and bi‐acetabular breadths were no longer significant. Birth weight remained a significant predictor of bi‐iliac breadth in the multiple regression model, however, model *R* ^2^ was reduced from 0.40 to 0.36.

This study builds on prior evidence, which suggested contributions of the early‐life environment to variability in adult pelvic morphology (Angel et al., 1987; Kelley & Angel, 1987; Nicholson, 1945; Thoms, 1936, 1947). It is, to our knowledge, the first attempt to formally situate the pelvis within DOHaD, and directly test for associations between proxies of fetal and postnatal growth and adult pelvic dimensions. Indeed, the pelvis has been understudied in the DOHaD literature, despite both its capacity to be shaped, like height, by early life nutritional conditions, and the potential importance of pelvis size and shape variability for risk of obstructed labor. In contrast, DOHaD has aided in understanding how early‐life nutritional and environmental exposures may impact tissue growth to increase the risk of chronic conditions like diabetes, cardiovascular disease, and obesity (Agarwal et al., 2018; Cameron & Demerath, 2002; McMillen & Robinson, 2005). Nutritional deprivation in utero, for example, was proposed to adversely impact pancreatic development, thus disrupting glucose homeostasis and predisposing to diabetes mellitus (Hales & Barker, 1992). Such a link requires an insult to occur within an early period of environmental sensitivity, or a so‐called “critical window” of phenotypic plasticity, which ultimately closes, resulting in the tracking of insult‐driven effects to adult phenotype (Barker, 1997; Fowden, Giussani, & Forhead, 2006; Godfrey & Barker, 2001).

The current study suggests, however, that variability in several pelvic dimensions may be sensitive to linear growth both in and after the first 2 to 3 years of postnatal life. Growth of the pelvis may broadly track growth in stature, despite adult height being achieved prior to the cessation of pelvic growth (Moerman, 1982; Sharma et al., 2016). Our findings do not confirm, but they are consistent, with the notion that an environmental insult in one growth period could be offset to some degree by improved conditions in another. This is captured, for example, by the phenomenon of “catch‐up growth,” which may occur in different growth periods following environmental insults (Cameron, 2012; Ong, Ahmed, Emmett, Preece, & Dunger, 2000); although, to reiterate, linear growth is relatively canalized following the first 2 to 3 years of life (Cameron, 2012; Lejarraga, 2012; Martorell, 1989). The promotion of early growth is clearly a priority, as the effects of poor nutrition on lower leg growth in the first two years of life contribute to risk for stunting (Cole, 2000a) and could plausibly result in failure to reach one's genetic potential for both height and the pelvis. This is supported by studies where shorter women were more likely to demonstrate a flat or contracted pelvis (Bernard, 1952; Cox, 1963; Kakoma, 2016), conditions that are associated with adverse birth and fetal growth outcomes (Kakoma, 2016; Martyn, Barker, & Osmond, 1996).

The populations represented in our study sample, those from Sri Lanka, Pakistan, India, and Bangladesh, ranked 29th, 16th, 9th, and 3rd from the bottom, respectively, in a recent survey of women's average height across 200 countries (NCD RisC, 2016). Some populations are struggling to reverse deficits in stature; for example, adult height in Bangladesh and India appears to have plateaued below trends in East Asia (NCD RisC, 2016). At the same time, and despite overall improvements in maternal mortality, 22% of ~300 000 maternal deaths worldwide occur in South Asia each year (Akseer et al., 2017). The maternal mortality ratio (maternal deaths per 100 000 live births) in 2015 was ~175 in Pakistan, Bangladesh, and India, and 30 in Sri Lanka (WHO, UNICEF, UNFPA, World Bank Group, & the United Nations Population Division, 2015), with obstructed labor, uterine rupture, and hemorrhage listed among the main causes of maternal death (Akseer et al., 2017). As mentioned in the introduction, evidence links variability in height to feto‐maternal disproportion and obstructed labor (Rush, 2000; Toh‐adam et al., 2012; Tsu, 1992), although height is clearly just one of many potential factors contributing to maternal mortality rates. For example, Guatemala, the shortest female population on average as recorded by NCD‐RisC (2016), had a maternal mortality ratio of 88 per 100 000 live births in 2015, and several countries in Africa with greater average height than those in South Asia recorded ratios >175 per 100 000 (WHO et al., 2015). Genetic covariance between height, head size, and pelvic shape may also play a role in alleviating feto‐maternal disproportion in some populations (Fischer & Mitteroecker, 2015; Kurki, 2007).

In India, evidence suggests that a negative secular trend in height over 10 000 years led to current low average height, possibly driven by transgenerational nutritional stresses, among other factors (Wells, Pomeroy, Walimbe, Popkin, & Yajnik, 2016). Importantly, secular height trends are principally influenced by postnatal growth (Cole, 2000a; Sanna & Soro, 2000), and our results are consistent with the suggestion that nutritional interventions to reduce stunting, already promoted for many other reasons (Dewey & Begum, 2011), might benefit both adult height and pelvis size. Prior work has examined secular trends in pelvic size and morphology, although results have been somewhat mixed. A 1982 study from Northern Ireland showed that secular increases in height were associated with secular increases in pelvic dimensions (Holland et al., 1982). Driscoll (2010), using skeletal material, identified changes in shape, but not size, of the female pelvis over time. Also examining skeletal collections, DelPrete (2006) reported evidence for alteration in pelvic morphology over three centuries, although changes were not uniform across the collections. This is an important area for further study as low‐ and middle‐income populations increasingly suffer from overweight and obesity, even as they continue to experience undernutrition, sometimes within the same household or individual (Doak, Adair, Bentley, Monteiro, & Popkin, 2005). A woman may, for example, be short and have a smaller pelvis due to trans‐generational or poor early nutritional circumstances and also experience overweight in adulthood as environmental exposures change. Overweight and obesity raise the risk of macrosomic offspring, increasing the risk of fetomaternal disproportion and obstructed labor. Indeed, a recent study found that Indian women who were both short and overweight were more likely to have a cesarean delivery than women who were either short or overweight alone (Wells, Wibaek, & Poullas, 2018).

The question of whether pelvimetry (measurement of the female pelvis) is useful for predicting obstructed labor or operative delivery at the individual level has been assessed by a number of authors, with mixed results (Connolly & McKenna, 2001; Harper, Odibo, Stamilio, & Macones, 2013; Lenhard et al., 2010; Liselele, Boulvain, Tshibangu, & Meuris, 2000; Spörri et al., 2002; Sun & Tong, 2014; Thubisi, Ebrahim, Moodley, & Shweni, 1993; Zaretsky et al., 2005). A recent Cochrane systematic review determined there was not enough evidence in the literature to support the use of X‐ray pelvimetry for predicting risk of obstructed labor and optimal mode of delivery (Pattinson, Cuthbert, & Vannevel, 2017). Moreover, it may be argued that access to adequate obstetric care plays a far greater role in determining rates of maternal/offspring morbidity and death due to obstructed labor (Campbell & Graham, 2006; Ronsmans et al., 2006). The current study recognizes these issues; however, it is important to look beyond predictions of birth outcomes at the individual level. Investigators must seek to characterize more broadly when, and how much, linear growth translates into pelvic growth, in order to understand how populations experiencing variable secular height trends and differential exposure to nutritional transition may be more or less susceptible to risk of obstructed labor.

A principal strength of this study is the use of three‐dimensional MRI, which offers the opportunity to measure the pelvis in vivo while avoiding potential confounding by overlying soft tissue. Moreover, all measurements were made on the same equipment by a single observer. However, the sample size was relatively small, and the precision of birth weight SDS may have suffered from poor recall, as mentioned above. We employed a cross‐sectional design and used proxies for fetal nutritional supply and early and later postnatal growth; longitudinal studies in diverse populations are needed to develop a clearer picture of how different growth periods shape adult pelvic phenotype. Finally, height (Lettre, 2011) and dimensions of the pelvis (Sharma, 2002) demonstrate heritability, and variability in human body proportions and size may also reflect long‐term differential exposure to ecological factors such as temperature (Ruff, 2010). Further studies are likewise needed to disentangle these effects from those related to nutritionally‐influenced developmental growth plasticity.

In conclusion, our study is consistent with others in demonstrating links between the pelvis and adult height, while also providing new evidence for potential positive effects of both early and later linear growth on adult pelvis size. At the same time, we found little evidence to support the prediction that in utero nutritional investment shapes pelvic dimensions. Future work should seek to characterize the developmental trajectory of different pelvic dimensions and also investigate the potential impacts of early‐life nutritional interventions (eg, those aimed at reducing stunting) on adult pelvis size and shape. It will be important to focus efforts on reversing negative secular trends in height, especially in populations where maternal BMI is increasing (Wells et al., 2018). This can be achieved by improving success in the prevention of early linear growth faltering and stunting.

## AUTHOR CONTRIBUTIONS

MKS and JCKW codesigned the study. MKS collected and analyzed the data and wrote the first draft of the manuscript. OJA assisted with data collection, data processing, and analysis. TJC served as statistician for the study. JCKW and CAC contributed to data acquisition and served as principal investigators. All authors provided critical comments on the manuscript and approved the final version.
